# Somatic mutational profiling identifies aggressive and indolent disease phenotypes in well-differentiated pancreatic neuroendocrine tumors

**DOI:** 10.3389/fonc.2026.1757796

**Published:** 2026-05-08

**Authors:** Leanne M. Brown, Ryan A. Hagenson, Jason M. Sheltzer, John W. Kunstman

**Affiliations:** 1 Department of Surgery, Yale School of Medicine, New Haven, CT, United States; 2 Department of Radiation Oncology, Stanford School of Medicine, Palo Alto, CA, United States

**Keywords:** aggressive disease, biomarker, pancreatic neuroendocrine tumor, PNET, TP53 mutant

## Abstract

**Introduction:**

Integration of biomarkers into clinical management of well-differentiated pancreatic neuroendocrine tumors (PNETs) remains limited due to disease rarity and heterogeneity. By utilizing publicly available genomic databases, we’ve established a large cohort of well-differentiated PNETs for study, focusing on somatic mutations and disease subgroup stratification through a modified targeted sequencing approach.

**Methods:**

A total of 434 patients, representing 310 primary tumors and 124 metastatic tumors, from nine publicly available genomic studies were included for study. A targeted sequencing panel consisting of 261 genes was applied across the cohort to establish somatic variant profiles for each patient. Patients were further allocated into established and novel molecular subgroups for additional investigation.

**Results:**

*MEN1* remains the most frequently mutated gene in both primary and metastatic tumors (41.9%, both). Within subgroup stratification, *ATRX* and *DAXX* mutations were not associated with a survival deficit in the metastatic setting, contrary to prior reports. *TP53* mutations, traditionally associated with neuroendocrine carcinoma, remained prevalent occurring in 5.5% and 19.4% of primary and metastatic tumors, respectively. An additional 4.2% of primary tumors and 9.7% of metastatic tumors harbor mutations in either *KRAS* or *SMAD4* that are more typically associated with pancreatic adenocarcinoma. *TP53* mutations were associated with metastasis, suggesting the presence of a more aggressive disease phenotype.

**Discussion:**

Somatic variants serve as prognostic biomarkers in well-differentiated PNETs. Profiling of *TP53*, *KRAS*, or *SMAD4* status may aid in identifying aggressive subtypes of disease. Additional evaluation of the utility of targeted tumor sequencing to guide PNET treatment is warranted.

## Introduction

Pancreatic neuroendocrine tumors (PNETs), a subset of a broad family of neuroendocrine neoplasms, are defined by their pancreatic origin and can be functional or nonfunctional depending on hormone secretion (1, 2). PNETs are biologically distinct from the more common pancreatic ductal adenocarcinoma; however, the incidence of PNETs is rapidly rising (3–5). Notably, these tumors are heterogeneous in presentation, behavior, and molecular features, resulting in significant challenges for clinical management. Treatment options for PNETs vary widely ranging from aggressive surgical resection, including metastatic disease, to a variety of systemic and local therapies (6–9). However, despite prior efforts in characterizing the molecular landscape of PNETs, there remains little application of these data to clinical practice.

More limited prior landscape studies investigating the somatic mutational profiles of PNETs at the whole genome and exome levels have demonstrated some recurrent mutational patterns. The most prevalent aberrations include alterations in chromatin remodeling (*MEN1, ARID1A*), telomere maintenance (*ATRX, DAXX*), DNA damage repair *(CHEK2, BRCA2)*, and the mTOR pathway (*PTEN, TSC1, TSC2*) (10–18). From these efforts, certain mutations appear to have prognostic significance and may potentially serve as biomarkers for selection of targeted therapies. Sequencing assays to detect these mutations are readily available in everyday clinical practice (19, 20). However, at present, integration of these molecular data to aid in clinical decision making is minimal. Instead, clinical management of PNETs is largely driven by resectability, differentiation status, and tumor grade (21). Well-differentiated tumors, comprising the vast majority of tumors, are molecularly and histologically distinct from poorly-differentiated pancreatic neuroendocrine carcinoma (PNEC), a rare, highly aggressive type (22–24). Well-differentiated PNETs are stratified by grade (1–3), defined by mitotic count and Ki-67 staining, and serves as a proxy for disease aggression (25, 26). This classification system does not employ molecular data and the utility of somatic mutations in defining clinical disease is unknown. Furthermore, comprehensive understanding of the interaction of somatic mutations with other molecular alterations (epigenetics, copy number alterations, microRNA) remains limited, particularly in the context of clinical behavior (27–29).

Existing molecular analyzes of PNET are often limited to a single institution or assay, thereby limiting the ability to infer clinical significance. As a result, the published frequencies of reported mutations can be highly variable; for instance, *MEN1* has been found mutated in 25-56% of PNETs (10, 11, 13, 30). The degree of prognostic importance conferred by these mutations is unclear. For instance, *ATRX* and *DAXX* loss, both present in approximately 10-20% of PNETs, have been found to be both positive or negative prognostic markers depending on the study (11, 31–34). Prior efforts to reconcile these findings into workable molecular signatures to assist PNET classification have proven challenging (17, 35, 36).

Publicly available datasets have helped remedy this problem in other rare tumor types by allowing broad representative analysis of large cohorts in aggregate (37, 38). Therefore, we sought to perform a comprehensive analysis of somatic mutational variants in PNETs using available reported landscapes in aggregate. We hypothesize that providing accurate frequency and penetrance of recurrent mutations in PNET would further facilitate future molecularly driven research. In particular, we sought to examine whether somatic mutational data could be utilized to characterize PNETs by previously described subgroup classification schemes and whether these have clinical relevance. We also hypothesize there will be prognostic implications of these typical manifestations and that routine somatic sequencing of PNETs could assist in clinical decision making.

## Materials and methods

### Data acquisition and development of patient and sample cohort

All available genomic repositories with PNET tumors on cBioPortal datahub (https://github.com/cBioPortal/datahub) were included for analysis. Mutational, sample, and patient data were downloaded from the selected datasets: PANET ARCNET 2017, PANET JHU 2011, MSK-IMPACT, MSK-MET, PANET MSK ERC 2023, PANET Shanghai, PCAWG, MET500, OrigiMed (11, 13, 39–43). Additional clinical information, including grade, differentiation status, and staging, was sourced from original publications if unavailable from cBioPortal; however, comprehensive histopathological information remained incomplete for the aggregated cohort. Samples included have a documented diagnosis of PNET or OncoTree code of “PANET”, a designation for well-differentiated disease. A summary of individual study nomenclature, histopathological, grade, differentiation, and staging information is available in **Supplementary Table 1**. Patients with documented neuroendocrine carcinoma or PNET Grade 3 were excluded from analysis. For patients with multiple lesions, the sample with the highest TMB (nonsynonymous) was selected for analysis as the representative sample. Samples included represent individual patients; paired tumor data was not included. Copy number alterations data remained incomplete and epigenetic data was absent, therefore not included within this study.

### Data harmonization and cleaning

Data was harmonized across cohorts by including all lesions with available somatic mutational data. As various sequencing approaches were utilized across studies the intersection of the MSK-IMPACT 341 and OrigiMed 450 gene panels was applied to the final aggregate tumor cohort for standardization of mutational data for subsequent analyzes (**Supplementary Table 1**) (39, 43). Additional subgroup analysis representing studies reporting whole exome sequencing (WES)/whole genome sequencing (WGS) results were performed for comprehensive review. Only nonsynonymous variants were included for analysis. The code used to perform this analysis is available at https://github.com/sheltzer-lab/PNET_somatic_mutations.

### Software versions

Analyses were performed using R (version 4.3.2) (44), PRISM (version 9.4.1), and Python (version 3.10.8) (45). R packages: car (version 3.1-2) (46), ggplot2 (version 3.4.4) (47), ggsurvfit (version 1.10) (48), gt (version 0.10.1) (49), gtsummary (version 1.7.2) (50), maftools (version 2.14.0) (51), openxlsx (version 4.2.5.2) (52), reshape (version 1.4.4) (53), survival (version 3.8-2) (54), tidyverse (version 2.0.0) (55). Python packages: matplotlib (version 3.6.2) (56), and pandas (version 1.5.2) (57).

### Tumor subgroup designation

In efforts to characterize distinct subsets of PNET disease, primary and metastatic tumors were allocated into molecular subgroups by two methods: one previously established classification method by *Ciobanu* et al. and one novel classification method proposed in this study. *Ciobanu* et al. subgrouping included “PanNEN1”, “PanNEN2”, “PanNEN4”, “PanNEN3/PanNEN5”. “PanNEN3/PanNEN5” represents PanNEN3 and PanNEN5 together, due to the lack of associated somatic variants to define individual groups. Novel subgrouping within this study included adaptation of *Ciobanu* et al. subgrouping with consideration for *TP53*, *KRAS*, and *SMAD4* mutations. Novel subgrouping included “*ADM*-Mutant” (representing *ATRX-*, *DAXX-*, and *MEN1*-mutant tumors), “*ADM*-WT” (representing ATRX-, DAXX-, MEN1-WT tumors), “*ADM*-WT”, “*ATRX/DAXX*-Mutant”, “*MEN1*-Mutant”, and “*TP53/KRAS/SMAD4*-Mutant”. Tumors with *TP53*, *KRAS*, or *SMAD4* mutations were designated as “*TP53/KRAS/SMAD4*-Mutant” regardless of the remaining somatic mutational profile. To be classified in a subgroup, specific somatic mutational signatures must be present within the tumor sample (**Supplementary Table 2**).

### Somatic mutational frequencies by tumor type and subgroup designation

To investigate somatic mutational landscape by lesion type and PNET subgroup classification, associated mutational frequencies were calculated. To exclude infrequently mutated genes, a somatic mutation must have been present in at least 5% of tumors included in lesion type and subgroup analyzes. Mutational frequencies were determined as proportion of samples with somatic mutation present over the total number of samples included within each subgroup analysis. Analyses included aggregated cohort by sample type and subgroup classifications by tumor type. Testing for association was done via Pearson’s Chi-squared test or Fisher’s exact test across lesion types in the aggregated cohort and subgrouping analyzes.

### Logistic regression of somatic variants associated with metastasis

To determine which somatic mutations correlated with metastasis, a multivariate logistic regression model was utilized. Genes mutated in at least 5% of primary or metastatic tumors were included for analysis. The model included removal of covariates in two stages: 1) removal of aliased covariates (i.e., those with perfect correlation to another covariate), and 2) recursive removal of the covariate with the highest variance inflation factor (VIF) until all covariates had a VIF ≤ 4. Clinical correlates, including known grade, differentiation status, and stage, remained incomplete for the aggregated cohort and therefore unable to be incorporated for analysis. Significance of covariates was tested by the Wald test followed by multiple hypothesis correction with Benjamini & Hochberg’s method (58).

### Survival analysis

Kaplan-Meier survival analysis of pooled overall survival was performed in R, using the packages: survival and ggsurvfit. Significance was tested via logrank test (59, 60).

### Data visualization

Graphs were generated using Graphpad Prism 10.1.0.

## Results

### Generation of a large, multi-institutional PNET cohort

To build a large, aggregate sample cohort, nine publicly available genomic datasets were accessed (11, 13, 39–43). Of these, 434 patients were identified, consisting of 310 (71.4%) primary tumors and 124 (28.6%) metastases; each tumor was unique with no paired primary and metastatic lesions analyzed. The total number of patients contributed per dataset varied, ranging from four PNET patients in MET500 to 157 patients in MSK-MET (40, 42). The majority of included patients originated from the United States (n=226, 52.1%) with 30.6% (n=133) of the patients from international cohorts in Asia, Europe, and Australia. Patients were predominantly reported as white (n= 259, 57.4%); however, 12.2% (n=55) and 4.0% (n=18) of patients identified as Asian and Black, respectively. There was a slight sex bias present, with 56.7% (n=246) of patients reported as male, similar to a recent SEER epidemiology report (61). Confirmed PANET oncocode designation, WHO grade, or differentiation status was reported for 85.3% of patients (n=370) but remained incomplete for the entire cohort. Reported differentiation status and grade was known in 52.7% of included samples (**Supplementary Table 1**). 84.6% (n=367) of patients had survival data accessible for analysis (**Table 1**). Information regarding familial syndromes was largely unknown.

**Table 1:** Patient demographics by lesion type.

Aggregated PNET samples				
		Primaries N=310	Metastases N=124	p-value
Race				<0.001
	Asian	29 (9.3%)	24 (19.4%)	
	Black	9 (2.9%)	9 (7.3%)	
	Pacific Islander	1 (0.0%)	0 (0%)	
	White	170 (54.8%)	76 (61.3%)	
	Other	3 (0.1%)	3 (2.4%)	
	Unknown	98 (31.6%)	12 (9.7%)	
Sex				0.408
	Female	129 (42.0%)	57 (46.0%)	
	Male	181 (58.0%)	67 (54.0%)	
Survival Status				<0.001
	Deceased	31 (10.0%)	36 (29.3%)	
	Living	237 (88.1%)	63 (50.8%)	
	Unknown	42 (13.5%)	25 (20.2%)	
Country				<0.001
	Australia	29 (9.4%)	0 (0%)	
	China	34 (11.0%)	19 (15.3%)	
	Italy	51 (16.5%)	0 (0%)	
	United States	121 (39.0%)	105 (84.5%)	
	Unknown	75 (24.2%)	0 (0%)	
Oncocode/Differentiation				0.021
	PANET/Well-differentiated	272 (87.8%)	98 (82.0%)	
	Unknown	38 (12.2%)	26 (18.0%)	
Original Study				
	MET500	0 (0%)	4 (3.2%)	
	MSK IMPACT	19 (6.1%)	16 (12.9%)	
	MSK-MET	76 (24.5%)	81 (65.3%)	
	Origimed 2020	24 (7.7%)	19 (15.3%)	
	PCAWG	75 (24.2%)	0 (0%)	
	PANET ARCNET	93 (30.0%)	0 (0%)	
	PANET JHU	7 (2.3%)	2 (1.6%)	
	MSK ERC 2023	6 (1.9%)	2 (1.6%)	
	Shanghai 2011	10 (3.2%)	0 (0%)	

Statistical testing includes Pearson’s Chi-squared and Fisher’s exact test. Acronyms: odds ratio (OR), confidence interval (CI). Acronyms: WHO (World Health Organization). Bold values indicate p < 0.05. The text size for the p-value of Oncocode/Differentiation is smaller then the other p-values.

### Primary and metastatic tumors demonstrate different somatic variant profiles

**Figure 1:**
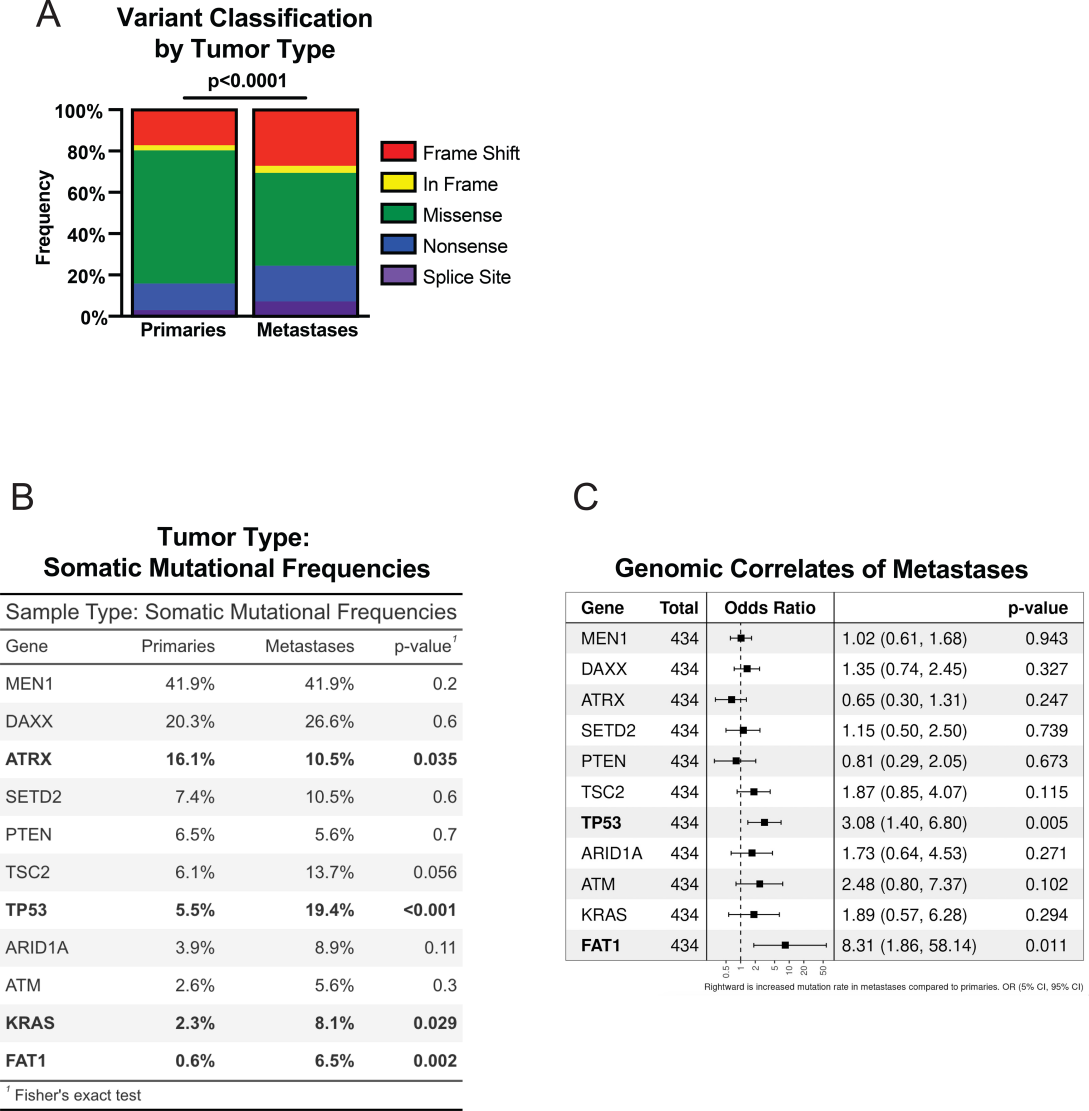
Variant profiling of primary and metastatic PNETs demonstrates recurrent somatic variants in primary and metastatic PNETs. **(A)** A bar graph demonstrating the distribution of somatic variant classification of primary and metastatic PNETs. **(B)** A table showing the most frequently mutated genes and mutational frequencies of primary and metastatic PNETs. **(C)** A table demonstrating the associations of somatic variants with the presence of metastases. Statistical testing was performed via two-tailed Pearson's Chi-squared test **(A, B)**, Wald test with correction by Benjamini-Hochberg's method **(C)**.

To assess the somatic variant landscape of PNET disease, we first sought to characterize the overall mutational profile associated by lesion type. As our cohort was representative of various sequencing modalities, we utilized an aggregate 261 gene panel for this analysis. This represented the maximum number of genes overlapping and therefore evaluable in all patients across all included studies (**Supplementary Table 2**). For all tumors, a total of 1,093 nonsynonymous mutations were identified, with a mean of two variants per tumor (IQR 1-4). The most common type of variant observed in both primary and metastatic tumors were missense mutations, observed in 44.7% and 64.1% of cases, respectively (**Figure 1A**).

To further investigate somatic variants associated with PNET disease, we next identified the mutations found in primary and metastatic tumors. In line with previous reports, primary tumors demonstrated frequent mutations in genes involved in chromatin remodeling (*MEN1*, *ATRX*, *DAXX*, *SETD2*) and mTOR pathway (*PTEN*, *TSC2*) (**Figure 1B**) (11, 13, 62–64). *MEN1* remained the most frequently mutated gene in both primary and metastatic tumors with similar mutational frequencies (41.9%, both). These mutations were also present within metastatic lesions, along with mutations in *ARID1A* (8.9%) and *ATM* (5.6%), additional chromatin remodeling and DNA damage repair genes, respectively. Metastases also demonstrated mutations in genes involved in cell signaling and differentiation (*FAT1*: 6.5%) and receptor tyrosine kinase (RTK) signaling pathways (*KRAS*: 8.1%). Notably, *TP53* mutations were significantly more frequent in metastatic tumors compared to primary tumors (19.4% vs. 5.5%, p<0.001) (**Figure 1B**).

**Figure 2:**
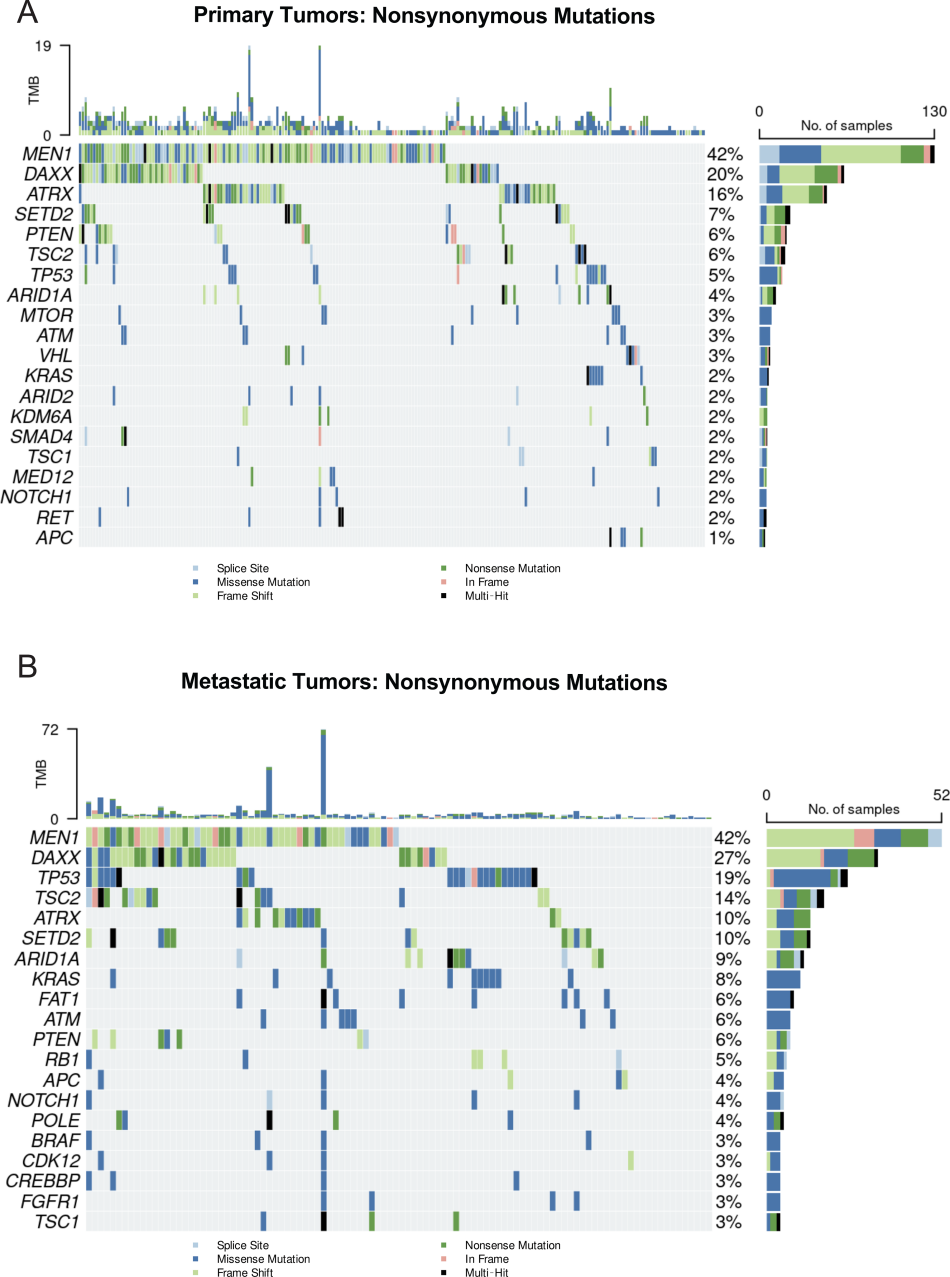
Primary and metastatic PNETs demonstrate different mutational profiles. **(A)** An oncoplot demonstrating the somatic mutational profiles of primary tumors. **(B)** An oncoplot demonstrating the somatic mutational profiles of metastatic tumors.

With the exception of *ATRX*, mutational frequencies of all epigenetic modifiers (*MEN1*, *DAXX, SETD2, ARID1A, ATM*) were similar between tumor types (p=NS, all). Similarly, the rate of *PTEN* mutations was similar between primary and metastatic tumors (6.5% vs. 5.6%, respectively). However, compared to primary tumors, metastases demonstrated significant increases in mutational frequencies of all remaining penetrant variants including *TP53*, *KRAS*, *FAT1*. Furthermore, a logistic regression model was built to further elaborate upon the mutational landscape differences by lesion type. Upon this analysis, *TP53* and *FAT1* mutations were significantly correlated with metastases (**Figure 1C**). We next created oncoprints to further define the somatic mutational profiles of both primary and metastatic tumors. Within primary tumors, *KRAS* mutations occurred in patients without *MEN1*, *ATRX*, or *DAXX* mutations (**Figure 2A**).

**Figure 3:**
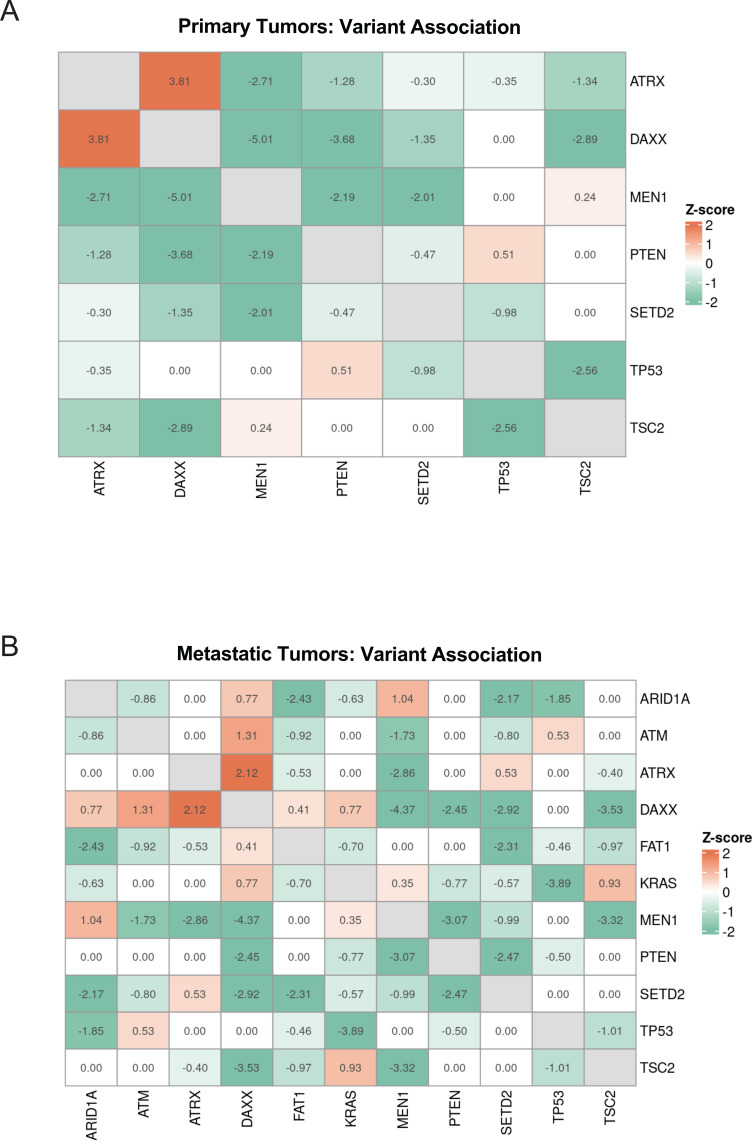
Primary and metastatic PNETs demonstrate different patterns of mutational variants. **(A)** A heat map demonstrating pairwise associations of the most common somatic variants of primary tumors. **(B)** A heat map demonstrating pairwise associations of the most common somatic variants of metastatic tumors. Statistical testing was performed via two-tailed Pearson’s Chi-squared test.

We next sought to delineate associations between penetrant variant pairs. Within primary tumors, *MEN1* demonstrated co-occurrence with *ATRX*, *DAXX, PTEN*, and *SETD2* mutations (z=-2.71, z=-5.01, z=-2.19, z=-2.01, respectively). As previously identified in prior landscape studies, *ATRX* and *DAXX* remained strongly mutually exclusive (z=3.81) (63). Notably, *DAXX* demonstrated additional patterns of co-occurrence with *TSC2* and *PTEN*, associations not present with *ATRX* mutations (**Figure 3A**). *ATRX* and *DAXX* remained mutual exclusive in metastatic tumors (z=2.12). *ARID1A* mutations were associated with the presence of several other mutations, *FAT1* and *SETD2* (z=-2.43, z=-2.17, respectively). Metastatic tumors also demonstrated a strong pattern of co-occurrence of *KRAS* and *TP53* mutations (z=-3.89) (**Figure 3B**).

### WES/WGS of PNET cohort demonstrate similar mutational frequencies to targeted sequencing in primary tumors

To assess whether the mutational burden and/or frequency of recurring mutated genes was meaningfully altered when assessed via targeted sequencing compared to agnostic assays, we sought to characterize the somatic mutational landscape within agnostic datasets alone for comprehensive review. 59.7% (n=185) of primary tumors underwent WES or WGS. Mutations in *TTN*, historically regarded as a passenger gene in cancer, was found to be present in 5.7% of primary tumors (65). Otherwise, WES/WGS did not demonstrate additional penetrant variants that were not identified through targeted sequencing. Within the WGS/WES cohort, primary lesions demonstrated similar mutational frequencies of *MEN1*, *ATRX*, and *DAXX* compared to the expanded targeted sequencing cohort (**Supplementary Table 4**). Metastases were excluded from analyzes given small sample size undergoing WES/WGS (4.8%, n=6).

### Application of established tumor subgrouping reveals clinically distinct patterns of PNET disease

**Figure 4:**
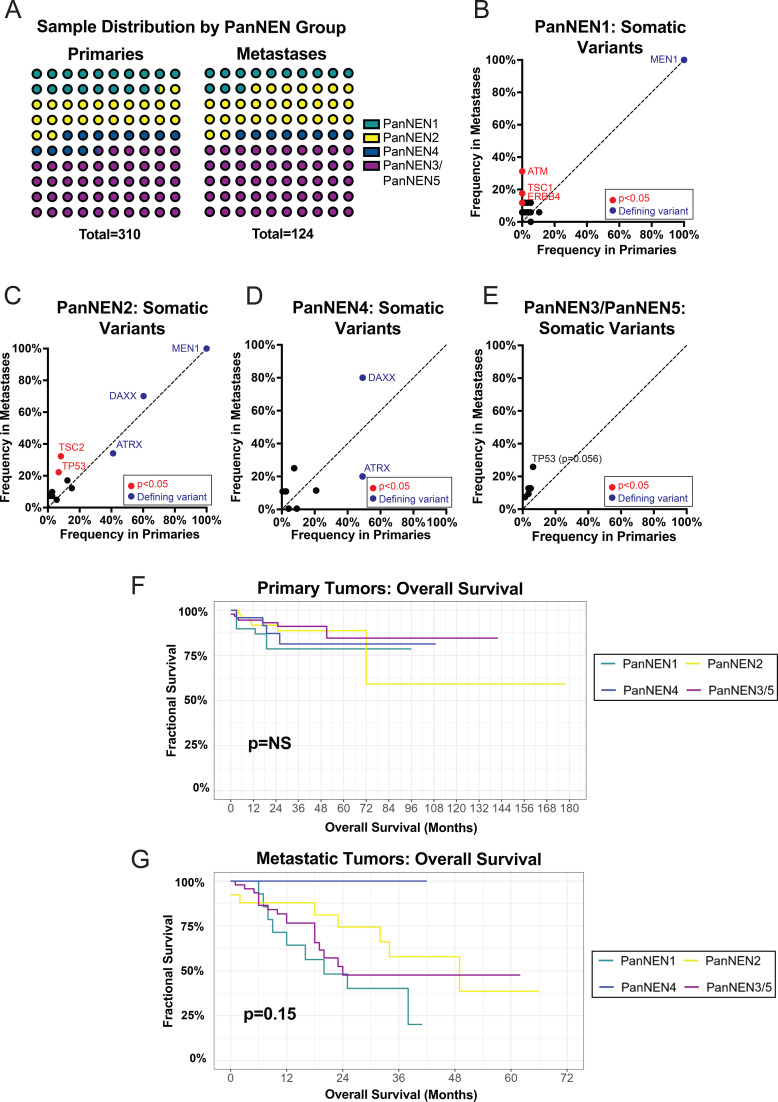
Stratification of PNETs by established somatic mutational profiles demonstrate distinct patterns of disease. **(A)** A figure displaying the distribution of PanNEN mutational subgroups in both primary and metastatic tumors. **(B)** A scatterplot demonstrating the frequencies of penetrant somatic variants in primary and metastatic tumors in the PanNEN1 subgroup. **(C)** A scatterplot demonstrating the frequencies of penetrant somatic variants in primary and metastatic tumors in the PanNEN2 subgroup. **(D)** A scatterplot demonstrating the frequencies of penetrant somatic variants in primary and metastatic tumors in the PanNEN4 subgroup. **(E)** A scatterplot demonstrating the frequencies of penetrant somatic variants in primary and metastatic tumors in the PanNEN3/5 subgroup. **(F)** A survival plot showing overall survival across the PanNEN mutational subgroups of primary tumors. **(G)** A survival plot showing overall survival across the PanNEN mutational subgroups of metastatic tumors. Statistical testing in B-E performed via Fisher's exact test, G-H performed via log-rank test. Acronyms: nonsignificant (NS).

To address the overall heterogeneity of PNETs, several prior groups have proposed subgroups of disease defined by both clinical and molecular characteristics (35, 36, 63). We applied subgroup labels established by *Ciobanu* et al., to our expanded cohort to define the remaining somatic landscapes within these subgroups by lesion type (**Figure 4A**, **Supplementary Table 3**) (35).

With stratification, clear patterns of associated mutational profiles are apparent by lesion type. PanNEN1 (*MEN1*-Mutant), a group representing non-functional, well differentiated NETs with a favorable prognosis, demonstrated frequent mutations in *ATM*, *ERBB4*, *TSC1*, found exclusively in metastatic lesions (**Figure 4B**). In contrast, PanNEN2 (*ATRX-* or *DAXX-*Mutant and *MEN1-*Mutant (*ADM*-Mutant) tumors), a group of non-functional tumors with high susceptibility to alternative lengthening of telomeres (ALT) activation associated with poor prognosis, demonstrated low incidences of *ATM* mutations in primary and metastatic tumors (5.5%, 0.0%, respectively); however, these tumors contained frequent TSC2 mutations (8.2% in primaries, 33.3% metastases, p=0.002), suggesting different molecular drivers between these tumor subgroups (**Figure 4C**). Within PanNEN4 (*ATRX-* or *DAXX-*Mutant), or the functional insulinoma subgroup high ALT susceptibility, metastases demonstrated similar mutational frequencies between primaries and metastases. Of note, *ATRX* and *DAXX* were mutated at similar frequencies in primary tumors (51.3% vs. 48.7%); however, metastases were enriched in *DAXX* mutations compared to *ATRX* mutations (80.0% vs. 20.0%) (**Figure 4D**). PanNEN3 (well-differentiated functional tumors) and PanNEN5 (heterogenous subgroup of nonfunctional well and poorly differentiated neuroendocrine neoplasms) were not associated mutational signatures in *Ciobanu* et al., thus these groups were combined and treated as one entity for analysis (35). As functional insulinomas remain rare in comparison to non-functional tumors, the PanNEN3/PanNEN5 group likely represents mostly PanNEN5 tumors (66, 67). PanNEN3/PanNEN5 tumors had fewer variants compared to other subgroups but demonstrated *TP53* mutations in both primary and metastatic lesions (6.4%, 24.2%, respectively) (**Figure 4E**). *TP53* mutations were frequent in PanNEN2, traditionally associated with a poorer prognosis, not previously described in *Ciobanu* et al. (35).

Comparative survival analysis between subgroups demonstrated prolonged survival in all patients with no difference in either primary or metastatic disease (**Figures 4F, G**). Within metastatic lesions, PanNEN1 and PanNEN3/PanNEN5 had the shortest median survival time of 20 and 24 months, respectively, compared to PanNEN2 (49 months), but these differences were not significant (**Figure 4G**).

### Identification of a PNET tumor subtype that harbors TP53/KRAS/SMAD4 mutations

Mutual exclusivity analysis demonstrated that *TP53* and *KRAS* frequently co-occurred with one another and without co-occurrence of the highest-penetrance mutations (*MEN1, ATRX, DAXX*) (**Figures 3A, B**). Tumors with *TP53* or *KRAS* mutations were distributed across nearly all PanNEN subgroups defined by *Ciobanu* et al. Notably, *TP53* and *KRAS* are the most frequently altered genes in pancreatic ductal adenocarcinoma (PDAC) or neuroendocrine carcinoma (PNEC), which are histologically and clinically distinct from well-differentiated PNET but are known to have a more aggressive clinical course (68, 69). A subset of PNETs was also found to harbor mutations in *SMAD4*, another highly altered gene in PDAC. PNETs with *TP53, KRAS*, or *SMAD4* mutations largely occurred without co-existing *MEN1* or *ATRX/DAXX* mutations and seemingly represented a unique genetic subgroup (**Figure 2B**).

**Figure 5:**
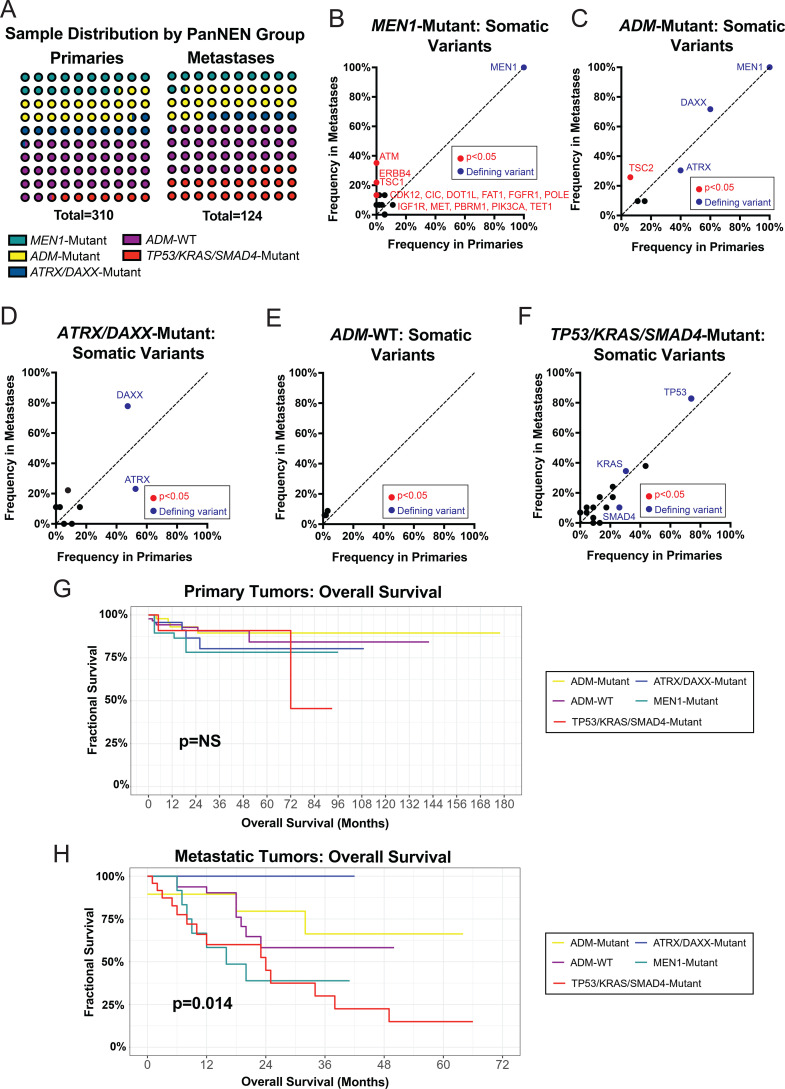
Expanded PNET subgrouping identifies a novel, high-risk disease with frequent *TP53/KRAS* mutations. **(A)** A figure displaying the distribution of mutational subgroups in both primary and metastatic tumors. **(B)** A scatterplot demonstrating the frequencies of penetrant somatic variants in primary and metastatic tumors in the *MEN1*-Mutant subgroup. **(C)** A scatterplot demonstrating the frequencies of penetrant somatic variants in primary and metastatic tumors in the ADM-Mutant subgroup. **(D)** A scatterplot demonstrating the frequencies of penetrant somatic variants in primary and metastatic tumors in the ATRX/DAXX-Mutant subgroup. **(E)** A scatterplot demonstrating the frequencies of penetrant somatic variants in primary and metastatic tumors in the *ADM*-WT subgroup. **(F)** A survival plot showing overall survival across the expanded PNET subgrouping of primary tumors. **(H)** A survival plot showing overall survival across the expanded PNET subgrouping of metastatic tumors. **(G)** A survival plot showing overall survival across the expanded PNET subgrouping of metastatic tumors. Statistical testing in B-E performed via Fisher's exact test, G-H performed via log-rank test. Acronyms: ADM (ATRX/DAXX/MEN1), WT (wildtype), NS (nonsignificant).

The existing classification schemes described above did not define those patients with *TP53/KRAS/SMAD4* mutations as a distinct group; thus, we defined a novel classification scheme with five categories, inclusive of a separate profile for tumors with these PDAC-like mutations. The presence or absence of *MEN1* or *ATRX/DAXX* mutations were utilized to define the other four categories (**Figure 5A**, **Supplementary Table 3**). This classification scheme was then applied to both primary and metastatic tumors, and survival analysis was performed (**Supplementary Table 3**).

Application of this novel scheme found that 23 primary tumors (7.4%) and 29 metastatic tumors (23.4%) were allocated to the novel *TP53/KRAS/SMAD4*-Mutant group that otherwise would have been distributed across *Ciobanu* et al. grouping (**Figure 5A**, **Supplementary Table 3**). Although tumor grade and differentiation status remains incomplete for the cohort, 57.7% (n=30) tumors had designated grade 1 disease, 7.7% (n=4) grade 2 disease, and 36.4% (n=18) remained unknown. Additional molecular tumor subgroups defined by *TP53/KRAS/SMAD4* wildtype status and variant presence of *MEN1* and *ATRX/DAXX* mutations demonstrated similar profiles within primaries and metastases to corresponding *Ciobanu* et al. groupings (**Figures 5B–E**). The novel *TP53/KRAS/SMAD4*-Mutant demonstrated similar mutational profiles between primaries and metastases (**Figure 5F**). In the survival analyzes, *TP53/KRAS/SMAD4*-Mutant patients demonstrated non-inferior overall survival in both localized and metastatic disease (**Figures 5G, H**). In patients with metastatic tumors, the presence of a mutation in *ATRX* or *DAXX* had the longest overall survival, although these differences did not reach statistical significance (**Figure 5H**). In this cohort of metastatic tumors, no patients with *ATRX/DAXX* mutations without a concomitant *MEN1* mutation died during the study period.

## Discussion

At present, clinical management of well-differentiated PNETs is dictated by symptomatic burden and tumor characteristics such as size and grade without incorporation of molecular biomarkers. Comprehensive understanding of PNET biology remains limited due to disease rarity and genomic characterization efforts often reflected single-institutional studies (11, 13). As tumor somatic sequencing becomes more commonly integrated into the diagnostic workup of rare tumors, evaluation of the utility of targeted gene panels in prognosticating PNET disease may have profound clinical impact. PNET is the ideal tumor type to apply aggregated large cohort analyzes across publicly-available genomic repositories. This study represents one of the largest aggregates of well-differentiated PNET disease utilizing publicly available genomic repositories in efforts to further characterize somatic variants underlying disease and define clinically-relevant subgroups.

We found that the mutational frequency of established high-frequency PNET variants (*MEN1, ATRX, DAXX)* remained similar to prior landscape studies (11–13, 59). Other known variants (*TSC2, PTEN, SETD2)* thought to influence disease were also present within our cohort. The large sample size here provides the most definitive prevalence of these variants within well-differentiated PNETs to date. Mutations in epigenetic modifiers and chromatin maintenance genes, including *MEN1, ATRX*, and *DAXX* remain prevalent in primary tumors. Importantly, these variants are similarly mutated within metastases; albeit *ATRX* and *DAXX* lose relatively mutual exclusivity suggesting these pathways may predominantly be implicated in early disease while possibly other epigenetic or variant aberrations may underlie metastatic spread. Metastatic lesions are more frequently *DAXX-*Mutant compared to *ATRX-*Mutant, suggesting that despite a shared molecular pathway in tumorigenesis, *DAXX* mutations may become more prominent as progression to metastatic disease occurs. Upon subgroup stratification, both primary and metastatic tumors with *ATRX* and/or *DAXX* mutations had non-inferior survivorship compared to other groups (**Figures 4**, **5**). This is contrary to previous reports (12, 33). Our findings suggest that *ATRX/DAXX*-Mutant may represent indolent disease, as there were no deaths among the metastatic group during the entire study period. Additional research is required further interrogate clinical associations of this group and to investigate if this subgroup may benefit from deviations of standard management, such as observation or therapeutic de-escalation rather than aggressive management. Additional investigation is needed to further elaborate on both the molecular and prognostic implications of *ATRX* and *DAXX* mutations.

*TP53*, *KRAS*, and *SMAD4* mutations are more traditionally associated with PDAC and PNEC and thought to be largely absent from PNET disease (22, 23, 68). Notably, within our expanded cohort, *TP53* and *KRAS* mutations were noted to be frequent. In particular, *TP53* mutations were common (primaries: 5.4%, metastases: 19.4%) and correlated with metastasis upon logistic regression analysis; however, these findings would further benefit from additional consideration of clinical factors (stage, grade, differentiation) for comprehensive analysis. The presence of *TP53*, *KRAS*, and *SMAD4* mutations here did not correlate with changes in overall survival despite their association with more aggressive pancreatic malignancies like PDAC. However, the survival analysis is limited due to significant study heterogeneity and incomplete survival data. Overall survival is also less informative than progression- or recurrence-free survival in well-differentiated PNET. Therefore, additional research is needed to validate these genomic findings and further establish clinicopathological associations. Historically, prior studies found a prevalence of *TP53* or *KRAS* mutations in 0-7% of well-differentiated PNETs (13, 70). Of note, a recent small cohort study demonstrated 35% of Grade 3 PNETs were *TP53*-mutant, in which these tumors were thought to have transformed into higher grade lesions (71). This current analysis noted a much higher prevalence of these variants than previously reported. However, a limitation of this study is the inability to confirm histology, as these tumors may represent mischaracterized well-differentiated disease and instead reflect Grade 3 tumors, NEC, or PDAC lesions. However, within our limited cohort, 65% of TP53/KRAS/SMAD4-mutant samples had documented Grade 1 or 2 disease, therefore suggesting that these mutations occur within well-differentiated disease. Additional research is needed to clarify these findings in a histologically verified, large volume cohort.

This large analysis of somatic mutations within localized and metastatic PNET notably found highly prevalent recurring mutations in a limited subset of genes. Some of these, such as *MEN1, ATRX, DAXX*, and other less frequently altered genes, have been reported in the past and this represents the most accurate frequencies of these variants at the population level. Interesting novel findings related to these variants were noted, including *DAXX* mutations being enriched in the metastatic setting over *ATRX* (which was not observed in primary tumors), and the absence of a negative survival impact of *ATRX/DAXX* mutations as had been previously reported. Other genetic findings were quite novel; in particular, a much-greater frequency of *TP53* and *KRAS* mutations were observed, with *TP53* mutations being substantially enriched in the metastatic population. The nature of the available datasets precluded substantial analysis of the clinical impact of these findings, yet their crucial role in the pathogenesis of many different cancer types encourages more granular correlative research in the future. The current study is limited by both the available clinicopathologic variables for analysis and the absence of other molecular aberrations present in these tumors. In particular, more relevant clinical endpoints such as recurrence or progression need to be assessed. An important finding of this study is that the number of recurring genes altered in well-differentiated PNET is small. Thus, future research could use narrow targeted panels to assess somatic mutations in PNETs and avoid more costly approaches. The role of mutational profiling in PNET clinical decision-making will remain limited, but future studies combining these data with more robust molecular analyzes beyond somatic variants may identify viable comprehensive biomarker panels to guide treatment.

## Data Availability

Publicly available datasets were analyzed in this study. This data can be found here: https://www.cbioportal.org/.

## Supplementary material

Supplementary Table 1 Individual dataset histopathological confirmation and nomenclature.

Supplementary Table 2 Shared sequenced genes between datasets as applied targeted gene panel.

Supplementary Table 3 PNET subgroup by somatic mutational profiling. **ATRX/DAXX* mutations are mutually exclusive. ADM, ATRX/DAXX/MEN1.

Supplementary Table 4 Somatic mutational frequencies in primary tumors.
