# Supplementary material for: Somatic mutational profiling identifies aggressive and indolent disease phenotypes in well-differentiated pancreatic neuroendocrine tumors
Source: Front Oncol. 2026 May 8;16:1757796. doi: 10.3389/fonc.2026.1757796 (PMC13193833; doi:10.3389/fonc.2026.1757796)
Supplement: Supplementary Table 1 — Individual dataset histopathological confirmation and nomenclature. [file Table1.docx]

**Table S1: Individual dataset histopathological confirmation and nomenclature**

| **Dataset** | **Histopathologic Diagnosis/**  **Classification Schema per Study** | **Grade** | **Differentiation** | **PANET Oncocode in Primary Source** | **Stage** |
| --- | --- | --- | --- | --- | --- |
| MET500 | Multiple sources of data including clinical history, documentation of primary diagnosis, pathologic review of tissue samples for histopathological diagnosis | Unknown | Unknown | Used | Known |
| MSK IMPACT | Tumor types were defined using institutional cancer type annotations derived from clinical pathology diagnoses | Incomplete | Incomplete | Used | Incomplete |
| MSK-MET | Tumor types were defined using institutional cancer type annotations derived from clinical pathology diagnoses | Incomplete | Incomplete | Used | Incomplete |
| MSK ERC 2023 | Tumor types were defined using institutional cancer type annotations derived from clinical pathology diagnoses | Incomplete | Incomplete | Used | Incomplete |
| PCAWG | Histologic classifications provided by contributing ICGC/TCGA cohorts | Known | Known | Not Used | Known |
| PANET ArcNET | Tissue histology/diagnosis was confirmed independently by pathologist with specific expertise in pancreatic diseases | Known | Known | Not Used | Known |
| OrigiMed 2020 | Tumor types were annotated according to an institutional classification system: OncoTree | Unknown | Unknown | Not Used | Incomplete |
| PANET JHU | Pathologically diagnosed PNET samples, PNEC excluded | Known | Known | Not Used | Known |
| Shanghai 2011 | Pathological diagnosis confirmed following resection | Unknown | Unknown | Not Used | NA - All benign insulinomas |
| Total Samples (n=571) |  | 57.2% | 57.2% | 57.4% | 72.3% |
| Includied Cohort (n=434) |  | 53.9% | 53.9% | 47.9% | 74.0% |
