# Supplementary material for: Somatic mutational profiling identifies aggressive and indolent disease phenotypes in well-differentiated pancreatic neuroendocrine tumors
Source: Front Oncol. 2026 May 8;16:1757796. doi: 10.3389/fonc.2026.1757796 (PMC13193833; doi:10.3389/fonc.2026.1757796)
Supplement: Supplementary Table 2 — Shared sequenced genes between datasets as applied targeted gene panel. [file Table2.docx]

**Table S2: Shared sequenced genes between datasets as applied targeted gene panel.**

| **Applied Shared Gene Panel (n=261)** | | | | | | | |
| --- | --- | --- | --- | --- | --- | --- | --- |
| *ABL1* | *CBFB* | *EPCAM* | *GNAQ* | *MDM4* | *PBRM1* | *RPTOR* | *TSC1* |
| *AKT1* | *CBL* | *EPHA3* | *GNAS* | *MED12* | *PDCD1* | *RUNX1* | *TSC2* |
| *AKT2* | *CCND1* | *EPHA5* | *GRIN2A* | *MEF2B* | *PDGFRA* | *SDHA* | *TSHR* |
| *AKT3* | *CCND2* | *EPHB1* | *GSK3B* | *MEN1* | *PDGFRB* | *SDHB* | *U2AF1* |
| *ALK* | *CCND3* | *ERBB2* | *HGF* | *MET* | *PIK3CA* | *SDHC* | *VHL* |
| *APC* | *CCNE1* | *ERBB3* | *HNF1A* | *MITF* | *PIK3CB* | *SDHD* | *WT1* |
| *AR* | *CD274* | *ERBB4* | *HRAS* | *MLH1* | *PIK3CD* | *SETD2* | *XIAP* |
| *ARAF* | *CD79B* | *ERG* | *IDH1* | *MPL* | *PIK3CG* | *SF3B1* | *XPO1* |
| *ARID1A* | *CDC73* | *ESR1* | *IDH2* | *MRE11A* | *PIK3R1* | *SMAD2* | *YES1* |
| *ARID1B* | *CDH1* | *ETV1* | *IGF1R* | *MSH2* | *PIK3R2* | *SMAD3* |  |
| *ARID2* | *CDK12* | *ETV6* | *IGF2* | *MSH6* | *PMS2* | *SMAD4* |  |
| *ASXL1* | *CDK4* | *EZH2* | *IKBKE* | *MTOR* | *POLE* | *SMARCA4* |  |
| *ATM* | *CDK6* | *FAM46C* | *IKZF1* | *MUTYH* | *PPP2R1A* | *SMARCB1* |  |
| *ATR* | *CDK8* | *FANCA* | *IL7R* | *MYC* | *PRDM1* | *SMARCD1* |  |
| *ATRX* | *CDKN1A* | *FANCC* | *INPP4B* | *MYCN* | *PRKAR1A* | *SMO* |  |
| *AURKA* | *CDKN1B* | *FAT1* | *IRF4* | *MYD88* | *PTCH1* | *SOCS1* |  |
| *AURKB* | *CDKN2A* | *FBXW7* | *IRS2* | *NBN* | *PTEN* | *SOX2* |  |
| *AXIN1* | *CDKN2B* | *FGF19* | *JAK1* | *NCOR1* | *PTPN11* | *SOX9* |  |
| *AXIN2* | *CDKN2C* | *FGF3* | *JAK2* | *NF1* | *RAC1* | *SPEN* |  |
| *AXL* | *CHEK1* | *FGF4* | *JAK3* | *NF2* | *RAD50* | *SPOP* |  |
| *BAP1* | *CHEK2* | *FGFR1* | *JUN* | *NFE2L2* | *RAD51* | *SRC* |  |
| *BARD1* | *CIC* | *FGFR2* | *KDM5A* | *NKX2-1* | *RAD51B* | *STAG2* |  |
| *BCL2* | *CREBBP* | *FGFR3* | *KDM5C* | *NOTCH1* | *RAD51C* | *STK11* |  |
| *BCL2L1* | *CRKL* | *FGFR4* | *KDM6A* | *NOTCH2* | *RAD51D* | *SUFU* |  |
| *BCL2L11* | *CRLF2* | *FH* | *KDR* | *NOTCH3* | *RAD52* | *SYK* |  |
| *BCL6* | *CSF1R* | *FLCN* | *KEAP1* | *NOTCH4* | *RAD54L* | *TBX3* |  |
| *BCOR* | *CTCF* | *FLT1* | *KIT* | *NPM1* | *RAF1* | *TERT* |  |
| *BLM* | *CTNNB1* | *FLT3* | *KRAS* | *NRAS* | *RARA* | *TET1* |  |
| *BMPR1A* | *CUL3* | *FLT4* | *LMO1* | *NSD1* | *RB1* | *TET2* |  |
| *BRAF* | *DAXX* | *FOXL2* | *MAP2K1* | *NTRK1* | *RBM10* | *TGFBR1* |  |
| *BRCA1* | *DDR2* | *FOXP1* | *MAP2K2* | *NTRK2* | *REL* | *TGFBR2* |  |
| *BRCA2* | *DICER1* | *FUBP1* | *MAP2K4* | *NTRK3* | *RET* | *TMPRSS2* |  |
| *BRD4* | *DNMT3A* | *GATA1* | *MAP3K1* | *PALB2* | *RHOA* | *TNFAIP3* |  |
| *BRIP1* | *DOT1L* | *GATA2* | *MAP3K13* | *PARK2* | *RICTOR* | *TNFRSF14* |  |
| *BTK* | *EGFR* | *GATA3* | *MCL1* | *PARP1* | *RNF43* | *TOP1* |  |
| *CARD11* | *EP300* | *GNA11* | *MDM2* | *PAX5* | *ROS1* | *TP53* |  |
