# Supplementary material for: Somatic mutational profiling identifies aggressive and indolent disease phenotypes in well-differentiated pancreatic neuroendocrine tumors
Source: Front Oncol. 2026 May 8;16:1757796. doi: 10.3389/fonc.2026.1757796 (PMC13193833; doi:10.3389/fonc.2026.1757796)
Supplement: Supplementary Table 3 — PNET subgroup by somatic mutational profiling. *ATRX/DAXX mutations are mutually exclusive. ADM, ATRX/DAXX/MEN1. [file Table3.docx]

**Table S3: PNET subgroup by somatic mutational profiling.**

| PNET Grouping by Somatic Mutational Profile | | | | | |
| --- | --- | --- | --- | --- | --- |
| Somatic Mutation | ***MEN1*-Mutant** | ***ADM*-Mutant** | ***ADM*-WT** | ***ATRX/DAXX*-Mutant*** | ***TP53/KRAS/SMAD4*-Mutant** |
| *MEN1* | + | + | - | - | +/- |
| *ATRX** | - | + | - | + | +/- |
| *DAXX** | - | + | - | + | +/- |
| *TP53/KRAS/SMAD4* | - | - | - | - | + |
| *Ciobanu et al.* Grouping | PanNEN1 | PanNEN2 | PanNEN3/PanNEN5 | PanNEN4 | NA |
| **ATRX/DAXX* mutations are mutually exclusive  Abbreviations: ADM: ATRX/DAXX/MEN1 | | | | | |
