# Supplementary material for: Somatic mutational profiling identifies aggressive and indolent disease phenotypes in well-differentiated pancreatic neuroendocrine tumors
Source: Front Oncol. 2026 May 8;16:1757796. doi: 10.3389/fonc.2026.1757796 (PMC13193833; doi:10.3389/fonc.2026.1757796)
Supplement: Supplementary Table 4 — Somatic mutational frequencies in primary tumors. [file Table4.docx]

**Table S4: Somatic mutational frequencies in primary tumors.**

| Gene | Targeted  Sequencing | WES/  WGS | p-value^1^ |
| --- | --- | --- | --- |
| *MEN1* | 45.7% | 37.2% | 0.6 |
| *DAXX* | 23.1% | 20.9% | 0.4 |
| *ATRX* | 16.9% | 11.5% | 0.7 |
| *SETD2* | 10.7% | 5.2% | 0.2 |
| *PTEN* | 5.4% | 7.3% | 0.15 |
| *TTN* | NA | 6.0% | NA |
| *TP53* | **15.6%** | **1.6%** | **<0.001** |
| *ARID1A* | **7.8%** | **2.1%** | **0.044** |
| *KRAS* | **7.0%** | **0.0%** | **<0.001** |
| ^1^Fisher’s exact test | | | |
